# Novel assays to assess the prevalence and neutralizing potential of anti-IdeS antibodies in healthy humans

**DOI:** 10.3389/fimmu.2026.1728855

**Published:** 2026-02-09

**Authors:** Emna Hannachi, Amélia Trecco, Victoria Daventure, Sandrine Delignat, Perrine Bonilla, Maxime Lecerf, Olivier Thaunat, Jordan D. Dimitrov, Claire Deligne, Sébastien Lacroix-Desmazes

**Affiliations:** 1 Institut National de la Santé et de la Recherche Médicale (INSERM), Centre de Recherche des Cordeliers, Centre National de la Recherche Scientifique (CNRS), Sorbonne Université, Université Paris Cité, Paris, France; 2 CIRI, Centre International de Recherche en Immunologie-infectiologie, Université de Lyon, INSERM U1111, Université Claude Bernard Lyon 1, CNRS, UMR5308, ENS de Lyon, Lyon, France

**Keywords:** anti-IdeS antibodies, IdeS, Imlifidase, neutralizing antibodies, synthetic FRET substrate, pre-existing immunity

## Abstract

**Background:**

The IgG-degrading enzyme IdeS (imlifidase) is a cysteine protease produced by *Streptococcus pyogenes*. It specifically hydrolyzes human IgG, cleaving the molecule to separate the F(ab’)_2_ fragment from the Fc region, thereby promoting IgG catabolism. The therapeutic form of IdeS (Idefirix^®^) is currently approved for use in patients undergoing kidney transplantation to eliminate donor-specific IgG, and in patients with Goodpasture syndrome to remove pathogenic anti-glomerular basement membrane antibodies. IgG antibodies directed against IdeS have been previously reported in both healthy individuals and kidney transplant recipients. However, the occurrence and potential clinical significance of anti-IdeS IgA antibodies and IdeS neutralizing antibodies have not been thoroughly investigated.

**Methods:**

In this study, we developed semi-quantitative enzyme-linked immunosorbent assays, synthesized a specific IdeS substrate, and validated a quantitative neutralization assay to detect and quantify anti-IdeS IgG, IgA, and IdeS neutralizing antibodies in healthy human plasma and serum samples.

**Results:**

We demonstrate the presence of anti-IdeS IgG capable of neutralizing IdeS enzymatic activity in therapeutic preparations of pooled normal human IgG (IVIg). Anti-IdeS IgG and IgA antibodies were detected in the plasma and serum of over 85% of 136 healthy individuals. However, clinically significant levels of IdeS-neutralizing activity were found in only ~1% of the individuals tested. IdeS-neutralizing activity was mediated exclusively by IgG, not IgA, and did not systematically correlate with levels of anti-IdeS IgG.

**Conclusions:**

Anti-IdeS IgG and IgA are highly prevalent in the normal population. This may relate to repeated infection by *S. pyogenes*. However, we found a low prevalence of clinically relevant levels of IdeS neutralizing antibodies. These findings highlight the need for a prospective clinical trial to assess IdeS-binding and IdeS-neutralizing antibody levels in kidney transplant recipients. Our novel functional IdeS neutralization assay offers a predictive tool to guide personalized medicine and determine patient eligibility for IdeS-based desensitization protocols.

## 1 Introduction

The IgG-degrading enzyme (IdeS) or imlifidase is a cysteine protease produced by *Streptococcus pyogens* (1, 2). IdeS sequentially hydrolyses the two heavy chains of the IgG between the hinge region and the CH2 domains between residues G236 and G237 in IgG1, IgG3 and IgG4, and between A236 and G237 in IgG2, thus dissociating the F(ab’)_2_ and the Fc fragment (3). Mutation of Cys^94^ in the active site of IdeS to a serine inactivates the proteinase (4). IdeS exhibits exquisite specificity for human IgG, but also hydrolyses rabbit and monkey IgG (5). Removal of the Fc fragment by IdeS drastically accelerates the catabolism of the F(ab’)_2_ of the IgG that are not recycled upon binding to the neonatal Fc receptor anymore (6–8). It also abrogates all Fc-dependent IgG functions, including Fcγ receptor-dependent phagocytosis, complement- and antibody-dependent cell-mediated cytotoxicity (9). The efficacy of IdeS in removing human IgG was first demonstrated in healthy individuals: complete IgG degradation was achieved in 2–6 hours for an IdeS dose of 0.24 mg/kg body weight (10). The therapeutic potential of IdeS (marketed as Idefirix^®^) has since been demonstrated in kidney transplant patients with donor specific antibodies (11) and in patients with Goodpasture syndrome (12, 13). It was also suggested in preclinical models of human diseases including rheumatoid arthritis (6, 12, 13), immune thrombocytopenic purpura (7), neuromyelitis optica (14) Tradtrantip, heparin-induced thrombocytopenia (15), hemophilia A (16, 17) as well as in the context of gene therapy (18).

The use of IdeS is however confronted by a pre-existing immunity that results from past infections by *S. pyogenes*. Indeed, increasing levels of anti-IdeS IgG were reported in patients with pharyngotonsillitis, bacteremia and erysipelas (19). The presence of anti-IdeS IgG was also reported in cohorts of healthy individuals (10) and in patients with chronic kidney disease (20). The existence of anti-IdeS IgA is however less clear. While infection by *S. pyogenes* occurs through the skin or mucosa, and should trigger mucosal immunity, the presence of anti-IdeS IgA in healthy donors has never been reported to our knowledge. We hypothesized that anti-IdeS IgG and/or IgA could alter the therapeutic efficacy of IdeS.

The neutralizing activity of anti-IdeS antibodies towards IdeS has been poorly investigated. Akesson et al. (19) detected IdeS neutralizing antibodies in the serum from patients with *S. pyogenes* infection: in one patient, IdeS-neutralizing activity was contained in the IgG fraction of the serum. In contrast, Johansson et al. (7) found that anti-IdeS antibodies in the serum from 19 healthy donors did not inactivate the IgG-cleaving activity of IdeS when used at a concentration that completely cleaves IgG in human blood *in vitro*. Pooled normal IgG for therapeutic use (IVIg) were however reported to contain IdeS neutralizing IgG (22). In agreement with this, IdeS was recently shown not to have the same efficacy in different patients undergoing kidney transplant, although no association with pre-existing IdeS neutralizing antibodies was investigated (23).

To our knowledge, there is to date no reliable quantification assay to measure anti-IdeS IgG, as most published work has relied on IVIg as a standard (10, 18), and there is a lack of a human anti-IdeS IgG standard. Besides, there is no sensitive, quantitative and high-throughput assay for the detection of IdeS neutralizing antibodies and most published works have used SDS-PAGE (7, 19, 22). In the present work, we addressed several objectives. First, we developed new assays to quantify anti-IdeS IgG and IgA in human serum and plasma. We further developed a high-throughput neutralization assay to measure IdeS neutralizing antibodies. Using these assays, we determined the prevalence of anti-IdeS IgG, anti-IdeS IgA and IdeS neutralizing antibodies among healthy blood donors.

## 2 Materials and methods

### 2.1 Source of antibodies and enzyme

BO2C11, KM41, BOIIB2 and LE2E9 are recombinant human IgG1 specific for factor VIII originating from patients with hemophilia A (17). Trastuzumab is a humanized monoclonal IgG specific for the human epidermal growth factor receptor 2 (Herceptin^®^, Roche). D02M03F05, D03M01D07 and D03M02C09 are recombinant human IgG1 originating from healthy individuals generated in our lab. Wild-type IdeS and the inactive IdeS^C94S^ variant were produced and purified as described (17). Intravenous immunoglobulins (IVIg) are pooled polyclonal IgG from healthy donors and were from Takeda Pharmaceuticals U.S.A., Inc. (Cambridge, MA).

### 2.2 Source of plasma/serum

Plasma (N = 80) and sera (N = 56) were prepared from blood of healthy donors (aged 18 to 85 years) collected from Etablissement Français du Sang Ile-de-France (C CPSL INSERM CENTRE EST – N°18/EFS/033). Samples numbers were empirical and based on availability at the start of the study, and are in line with a previous clinical study in healthy donors (10). The samples were kept at -80°C until use with minimal cycles of thawing and refreezing.

### 2.3 Cloning of anti-IdeS IgG^cl29^ and IgA^cl29^

B-cell hybridomas were generated by Proteogenix (Schiltigheim, France) following immunization of Balb/c mice with IdeS in Freund’s adjuvant and clone 29 was selected for the highest production of anti-IdeS IgG. The VH and VL genes of clone 29 were sequenced. The cDNA was cloned in human IgG and IgA expression vectors as described (24). In the case of the recombinant IgG_1_, the human hinge region was replaced by that of mouse IgG_1_ to prevent cleavage by IdeS. The recombinant humanized anti-IdeS IgG^cl29^ and IgA^cl29^ were produced in HEK293 cells using the Expi293™ Expression System (Thermofisher). IgG^cl29^ was purified from supernatant on protein A-coupled beads (GE Healthcare Life Sciences, Cytiva). IgA^cl29^ was purified on jacaline-agarose beads (Thermo Scientific Pierce). Purity was assessed by SDS-PAGE and concentrations determined by nanodrop (NanoDrop™, Thermo Scientific) with extinction coefficients of 235320 M^−1^cm^−1^ and 152640 M^−1^cm^−1^, respectively.

### 2.4 Anti-IdeS antibody binding enzyme-linked immunosorbent assay

ELISA plates (Maxisorp, Nunc) were coated with the inactive IdeS^C94S^ variant at 2.5 μg/ml in phosphate buffer saline (PBS, Sigma-Aldrich) overnight at 4°C, and blocked for 1 hour at 37°C using PBS-3% bovine serum albumin (BSA) or PBS-3% skim milk for detection of IgG or IgA, respectively. IVIg, human plasma, human sera, human monoclonal IgG, anti-IdeS IgG^cl29^ or IgA^cl29^, were then incubated in serial dilutions for an additional hour at 37°C. Bound human IgG were detected using a horseradish peroxidase (HRP)-conjugated mouse anti-human IgG Fc (9040-05, Southern Biotech), revealed using the peroxidase OPD substrate (Sigma-Aldrich) and optical densities (OD) were measured at 492 nm. Bound IgA were detected using an HRP-conjugated goat polyclonal anti-human IgA antibody (2050-05, Southern Biotech), revealed using the 3,3′,5,5′-tetramethylbenzidine (TMB) substrate (Sigma-Aldrich) and OD were measured at 450 nm. For competition ELISA, IVIg, IgG^cl29^ and donors’ plasma/serum were pre-incubated with increasing concentrations of IdeS^C94S^ in PBS-3% BSA for 1 hour at 37°C, prior to addition to the IdeS^C94S^-coated ELISA plates.

When indicated, IgG^cl29^ or IgA^cl29^ were used as standards to quantify anti-IdeS IgG or IgA in human plasma/serum, respectively. The limits of blank (LOB) for the quantitative IgG and IgA ELISAs were calculated using the OD of 20 randomly selected blanks (signal from wells with blocking buffer alone) (25), as LOB = mean_blank_ + 1.645 x SD_blank_, where SD represents the standard deviation. The limits of detection (LOD) were then estimated using the 20 samples with the lowest IgG or IgA concentrations (20Low), as LOD = LOB + 1.645 x SD_20Low_. The limits of quantification (LOQ) were determined using the 20 samples with the lowest IgG or IgA concentrations ≥ LOD (20LOD), as LOQ = LOB + 1.645 x SD_20LOD_. LOQ values for anti-IdeS IgG and IgA ELISAs are depicted as dotted lines on all respective graphs.

### 2.5 Digestion of BO2C11 and IVIg by IdeS

For *in vitro* cleavage, IVIg and BO2C11 (2000 nM each) were incubated in PBS alone or with IdeS (1–8 nM) for 6 hours at 37°C, at pH 7.4. For competitive digestion experiment, IVIg and BO2C11 (2000 nM) were incubated with 1 nM IdeS^C94S^ for 1 hour at 37°C in PBS pH 7.4, prior to the addition of 0. nM IdeS for an additional 6 hours. Samples were analyzed by SDS-PAGE using 12% gradient Bis-Tris protein Gel (NuPAGE, Thermosfisher) under non-reducing conditions followed by a coloration with Coomassie Blue.

### 2.6 Cloning of a synthetic IdeS substrate for fluorescence resonance energy transfer

The coding sequences for enhanced cyan fluorescent protein (eCFP) and Venus were obtained from Addgene (#105293 and #39813, respectively). The coding sequence for the hinge-CH2-CH3 of human IgG1 was obtained from Lecerf et al. (24). The gene encoding eCFP was fused with that encoding the Fc portion of human IgG1 from residue A231, containing two mutations at E356K and D399K. The gene encoding Venus was fused with that encoding the Fc portion of human IgG1 from residue A231, containing two mutations at K392D and K409D. The introduced mutations favor the heterodimerization of the eCFP-Fc/Venus-Fc construct by virtue of electrostatic steering (26). A GGGS linker was added between each fluorescent protein and the Fc fragment. A 6-his tag was added at the C-terminus of the eCFP-Fc monomer. The two cDNA were cloned independently in the pALL eukaryote expression vector under the control of the CMV promoter and using the murine IgG1 signal peptide (GenBank Accession Number DQ407610). The FRET substrate was expressed following co-transfection of HEK293 cells using the Expi293™ Expression System (Thermofisher). Occasionally, cells were transfected with each independent vector alone. The Venus-Fc expressed alone was purified on protein A-agarose (Cytiva). To this end, the supernatant was mixed with binding buffer (20 mM Na_2_HPO_4_, 150 mM NaCl pH 7). Venus-Fc was eluted with 0.1 M citric acid pH 3, neutralized with 1 M Tris pH 9. The eCFP-Fc/Venus-Fc FRET substrate and eCFP-Fc expressed alone were purified by immobilized metal ion chromatography. The supernatants were mixed with binding buffer (20 mM Na_2_HPO_4_, 150 mM NaCl, 20 mM imidazole pH 7) before purification and the recombinant proteins were eluted with elution buffer (20 mM Na_2_HPO_4_, 150 mM NaCl, 500 mM imidazole pH 7). All proteins were finally purified by size exclusion chromatography using Superdex 200 column against PBS to obtain only the heterodimer forms of the substrate or the monomeric form of the controls. The absorbance of the purified proteins was measured using a UV-Vis spectrophotometer (Agilent Technologies).

### 2.7 Digestion of the synthetic FRET substrate by IdeS

For dose-dependent digestion experiments, IdeS (0-4 µM) was incubated with the FRET substrate (2 µM) in PBS at 37°C for 45 min. For time-dependent digestion assays, IdeS (0.16 µM) and the FRET substrate (2 µM) were incubated in PBS at 37°C for 0, 5, 10, 20, 40, 80, 360 and 1440 minutes. Samples were then analyzed by SDS-PAGE 4-12% Bis-tris. When indicated, the emission spectra were recorded after excitation at 434 nm with a TECAN Infinite M200 Pro (Infinite^®^, Tecan Trading AG, Switzerland).

For determination of the kinetic parameters that define the hydrolysis of the FRET substrate by IdeS, the fluorescence ratio F_530 nm_/F_484 nm_ was calculated for the undigested substrate (R_0_) and for the substrate incubated with 1 nM IdeS 24 hours at 37°C (i.e., fully digested substrate, R_24_) following excitation at 434 nm. The FRET substrate (15 to 0.0625 µM) was then incubated with 1 nM IdeS for up to 380 sec with a reading of the fluorescence at 530 and 484 nm every 20 seconds. At each time point and for each substrate concentration, the fluorescence ratios F_530 nm_/F_484 nm_ (R) were calculated. The concentrations of digested substrate were finally calculated using the ratiometric FRET analysis method as described in Liu et al. (27):

C=(R-R_0_)/(R_24_-R_0_)xCi,

where Ci is the initial FRET substrate concentration. The initial velocity V_0_ (µM/sec) was computed during the linear phase by using the fluorescence R ratio measured at 380 seconds. The K_m_ and V_max_ were calculated following plotting of the V_0_ as a function of the substrate concentration, and fitting the experimental data to the Michaelis-Menten equation (Prism, version 9.4.1).

### 2.8 IdeS neutralization assay

IdeS (8–24 nM) was pre-incubated alone, or with serial dilutions of plasma/serum, IVIg or purified IgG, in PBS-0.025% Tween 20, pH 7.4 for 1 hour at 37°C in MicroWell™ 96 well polypropylene black plates (Nunc). The FRET substrate (500 nM) was then added for 1 hour at 37°C in the dark. The reaction was stopped by addition of 20 mM iodoacetamide and incubation for 30 min at room temperature in the dark. The “residual IdeS activity” was calculated as the ratio of measured fluorescence ratio F_530 nm_/F_484 nm_ for each condition over the fluorescence ratio measured in the absence of IdeS multiplied by 100. The percentage of IdeS neutralization was calculated as 100-”residual IdeS activity”.

The “clinically relevant IgG/IdeS ratio” used in the FRET IdeS neutralization assay was defined based on the available pharmacokinetic studies of therapeutic IdeS. Treatment of patients with therapeutic IdeS at 0.25 mg/kg (11, 13, 21, 28) yields a Cmax at the end of the injection of IdeS of 8.3 ± 3.7 µg/ml (10) (224 ± 100 nM, IdeS: 37 kDa). Hence, with a mean physiological concentration of circulating IgG of 13 mg/ml, the “clinically relevant IgG/IdeS ratio” corresponds to 13 mg/ml IgG for 224 nM IdeS. Accordingly, in the FRET neutralization assay, the “clinically relevant IgG/IdeS ratio” is reached when testing the serum or plasma at a 1:10 dilution with 24 nM IdeS.

### 2.9 Purification of IgG from plasma/serum

For IgG purification, plasma/serum (25 µl) was diluted 1:10 in purification buffer (Pierce™ Melon™ Gel IgG Spin Purification Kit) and incubated with 100 µl of Melon Gel for 10 min at room temperature. Purified IgG was collected. For IgG depletion, plasma/serum samples (25 µl) were diluted 1:10 in PBS prior to incubation with 100 µl Protein A-coupled agarose beads (Pierce Protein A/G Agarose) for 20 min at room temperature. The IgG-depleted flow-through was collected by centrifugation. Samples were stored at -20°C until use. IgG was quantified in plasma/serum, in the purified IgG fraction and in the IgG-depleted flow through by ELISA using an unlabeled goat anti-human Ig kappa antibody (2.5 µg/mL; 2060–01 Southern Biotech) for capture, and an HRP-conjugated mouse anti-human IgG Fc (1/3000, 9040–05 Southern Biotech) for detection. A standard curve was established using serial dilutions of IVIg.

### 2.10 Statistical analysis

Data were analyzed using GraphPad Prism Software (San Diego, CA, USA). Outliers in both ELISAs and neutralization assay were retested at greater plasma/serum dilutions. The data were considered as not normally distributed. Statistical differences were assessed using the two-tailed non-parametric Mann-Whitney test. Correlations were assessed by two-tailed non-parametric Spearman correlation. P values below 0.05 were considered as statistically significant. Graphs depict data as means ± standard deviations, or as boxes and whiskers with the box depicting the median and 25th to 75th percentiles, and the whiskers depicting the min and max values.

## 3 Results

### 3.1 IdeS-neutralizing IgG are present in IVIg

**Figure 1:**
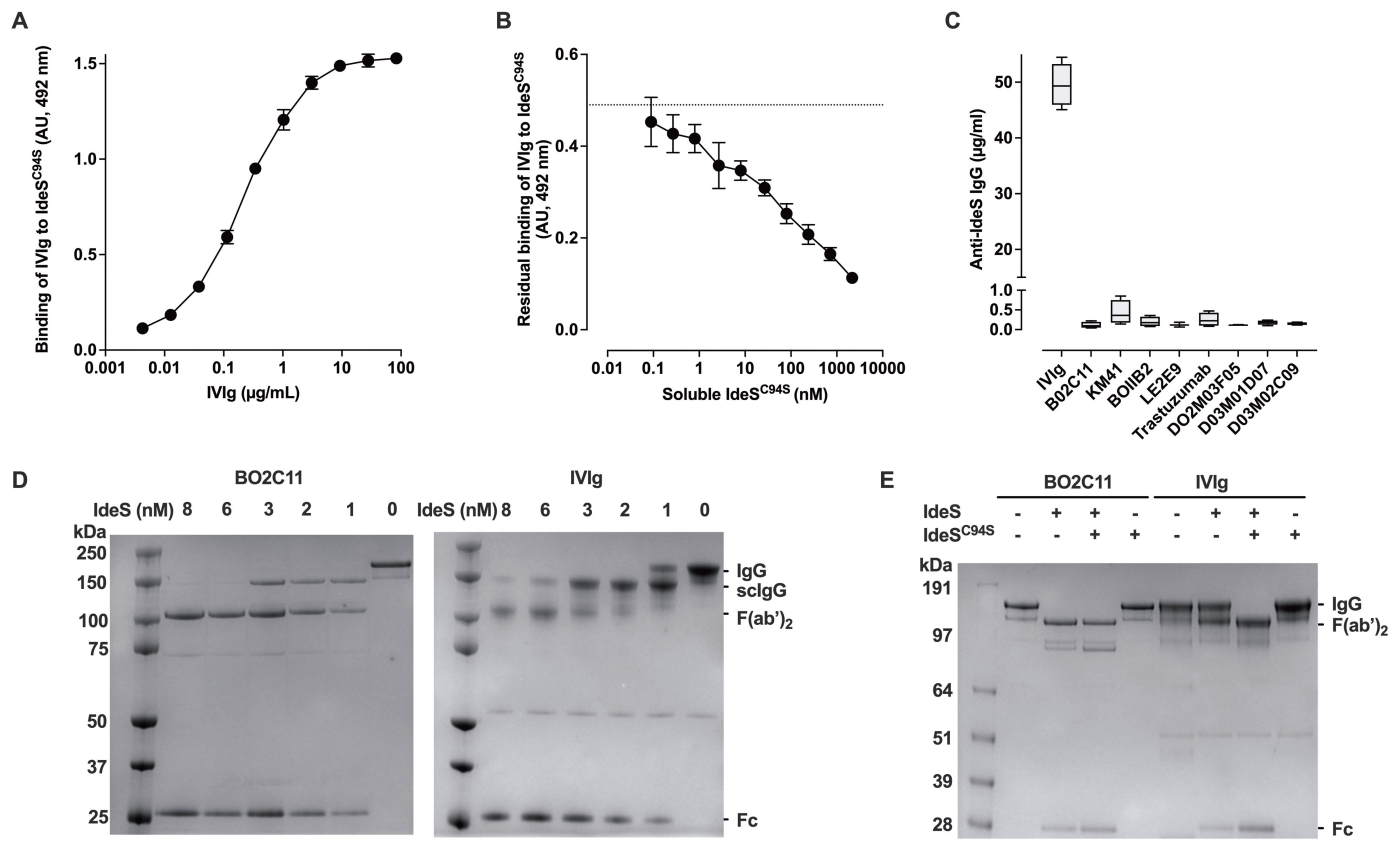
IVIg contains IgG that neutralize IdeS. **(A)** Anti-IdeS IgG in IVIg. The binding of IVIg to immobilized IdeS^C94S^ was detected by ELISA. Results are expressed in arbitrary units (AU) using the optical densities measured at 492 nm, and are depicted as mean ± standard deviation (SD) from 2 independent experiments. **(B)** Competitive inhibition of IgG binding to IdeS. IVIg (2.5 µg/ml) was incubated alone or with varying concentrations of IdeS^C94S^ prior to addition to IdeS^C94S^-coated plates. Results depict the residual binding of IgG to IdeS^C94S^ expressed in AU as mean ± SD from 2 independent experiments. **(C)** Absence of binding to IdeS of human monoclonal IgG1 not specific for IdeS. IVIg, and monoclonal human IgG1 (BO2C11, KM41, BOIIB2 and LE2E9, Trastuzumab, D02M03F05, D03M01D07 and D03M02C09) were incubated in serial dilutions on IdeS^C94S^-coated plates. Results are expressed as levels of anti-IdeS IgG (mean ± SD, n=5) determined using a humanized monoclonal anti-IdeS IgG^cl29^ (see **Figure 4**) as a standard. **(D)** *In vitro* cleavage of IVIg and BO2C11 by IdeS. IVIg and BO2C11 (2 µM) were incubated with IdeS (0, 1, 2, 3, 6 and 8 nM) for 6 hours at 37°C in PBS. Samples (2 μg/lane) were then separated by 12% SDS-PAGE under non-reducing conditions. Molecular weight markers are shown on the left of the gels. The protein bands corresponding to intact IgG, single-cleaved intermediate IgG (scIgG), F(ab’)_2_ and Fc fragments are shown on the right of the gels. **(E)** Competitive inhibition of IgG digestion by IdeS^C94S^. IVIg and BO2C11 (2 µM) were incubated with 1 nM IdeS^C94S^ for 1 hour at 37°C in PBS, prior to the addition of 0.5 nM IdeS for an additional 6 hours. Samples were analyzed by 12% SDS-PAGE under non-reducing conditions.

We first confirmed the presence of anti-IdeS IgG in IVIg, a pool of normal human IgG. IVIg bound in a dose-dependent manner to the immobilized inactive IdeS^C94S^ variant by ELISA (**Figure 1A**). The binding of IVIg to immobilized IdeS^C94S^ was inhibited in a dose-dependent manner by soluble IdeS^C94S^ with an IC_50_ of 108.6 ± 39.8 nM (**Figure 1B**). As controls, 7 human monoclonal IgG_1_ that are not specific for IdeS exhibited no binding to IdeS (**Figure 1C**), indicating that the binding of IVIg to IdeS^C94S^ occurs through the Fab and not through the hinge-CH2CH3 domains in a substrate-like manner (29). We then examined the hydrolysis of IVIg by IdeS. We first used BO2C11, one of the human monoclonal IgG, as a control. BO2C11 IgG was entirely hydrolyzed into scIgG and F(ab’)_2_ fragments after 6 hours at 37°C in the presence of 1 nM IdeS, the lowest concentration tested (**Figure 1D**, left panel, see **Supplementary Figure S1** for original uncropped gel images). The scIgG was undetectable in the presence of 6 nM IdeS, indicating complete hydrolysis. In contrast, the hydrolysis of IVIg was incomplete in the presence of 1 nM IdeS, with a residual band of intact IgG and no generation of F(ab’)_2_ (**Figure 1D**, right panel). Residual scIgG was still detectable after incubation in the presence of 8 nM IdeS. The complete hydrolysis of IVIg by 1 nM IdeS was however restored when IVIg was preincubated with IdeS^C94S^ prior to addition of IdeS (**Figure 1E**), suggesting saturation of the neutralizing anti-IdeS IgG fraction within IVIg. Conversely, addition of IdeS^C94S^ had no effect on IdeS-mediated BO2C11 hydrolysis. Taken together, the data indicate that IVIg contains anti-IdeS IgG and that the latter are able to neutralize the proteolytic activity of IdeS.

**Figure 2:**
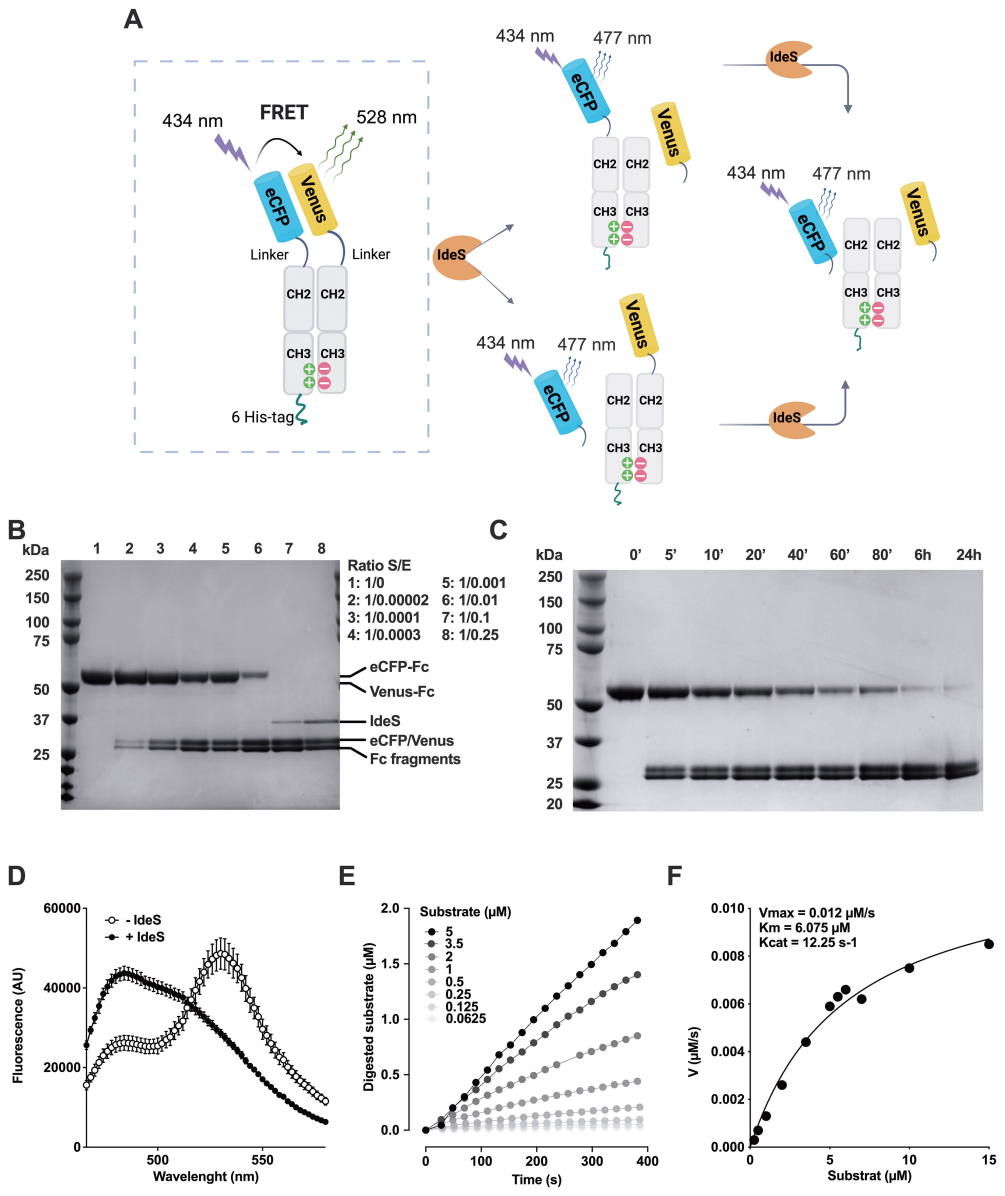
Validation of a synthetic FRET substrate. **(A)** Schematic representation of the FRET substrate. The FRET substrate combines the eCFP and Venus fluorochromes, each fused to the hinge region, CH2 and CH3 domains of the human IgG1 Fcγ. Heterodimerization is ensured by mutations in the CH3 domains conferring electrostatic steering (26). In the complete molecule, excitation of eCFP at 434 nm leads to the excitation of Venus and emission of fluorescence at 528 nm. When the substrate is cleaved by IdeS, on either chain or on both chains, the FRET signal is lost and excitation of eCFP at 434 nm results in emission signal at 477 nm. The ratio of fluorescence measured at 530 and 484 nm is used to quantify substrate cleavage. **(B, C)** Dose and time-dependent cleavage of the FRET substrate by IdeS. The FRET substrate (2 µM) was incubated in PBS at 37°C alone or with IdeS (up to 2 µM) for 45 min **(B)** or with IdeS (0.16 µM) for 0, 5, 10, 20, 40, 60, 80, 360 or 1440 min **(C)**. Proteins (2 µg/lane) were then separated by 12% SDS-PAGE. On both gels [but labeled only for panel **(B)**], the upper and lower bands of the top doublet correspond to the undigested eCFP-Fc-6His tag monomer (465 aa) Venus-Fc monomer (459 aa), respectively. In the bottom doublet, the upper and lower bands correspond to the digested eCFP/Venus (248 aa) and digested Fc/Fc-His tag fragments (211 or 217 aa), respectively. **(D)** End-point cleavage of the FRET substrate by IdeS. The FRET substrate (2 µM) was incubated alone (empty circles) or with IdeS (0.16 µM, full circles) in PBS for 24 hours at 37°C. The fluorescence emission profiles were measured following excitation at 434 nm. **(E)** Kinetics of IdeS-mediated FRET substrate cleavage. The FRET substrate (5-0.0625 µM) was incubated with 1 nM IdeS for up to 400 sec with excitations at 434 nm and fluorescence reading at 530 and 484 nm every 20 seconds. The concentration of digested substrate was calculated for each time point and each substrate concentration, and was plotted as a function of time, for each substrate concentration. Representative of two independent experiments. **(F)** Kinetic parameters of IdeS-mediated FRET substrate cleavage. The initial hydrolysis rates V_0_ (µM/s) were calculated for each substrate concentration (0.0625-15 µM), using the linear phase of the curves depicting the concentration of hydrolyzed substrate as a function of time **(E)**. The graph depicts the V_0_ (µM/s) plotted as a function of the substrate concentration. Experimental data were fitted to the Michaelis-Menten equation to derive the kinetic parameters. Data are pooled from two independent experiments. The derived kinetic parameters are shown in the graph.

**Figure 3:**
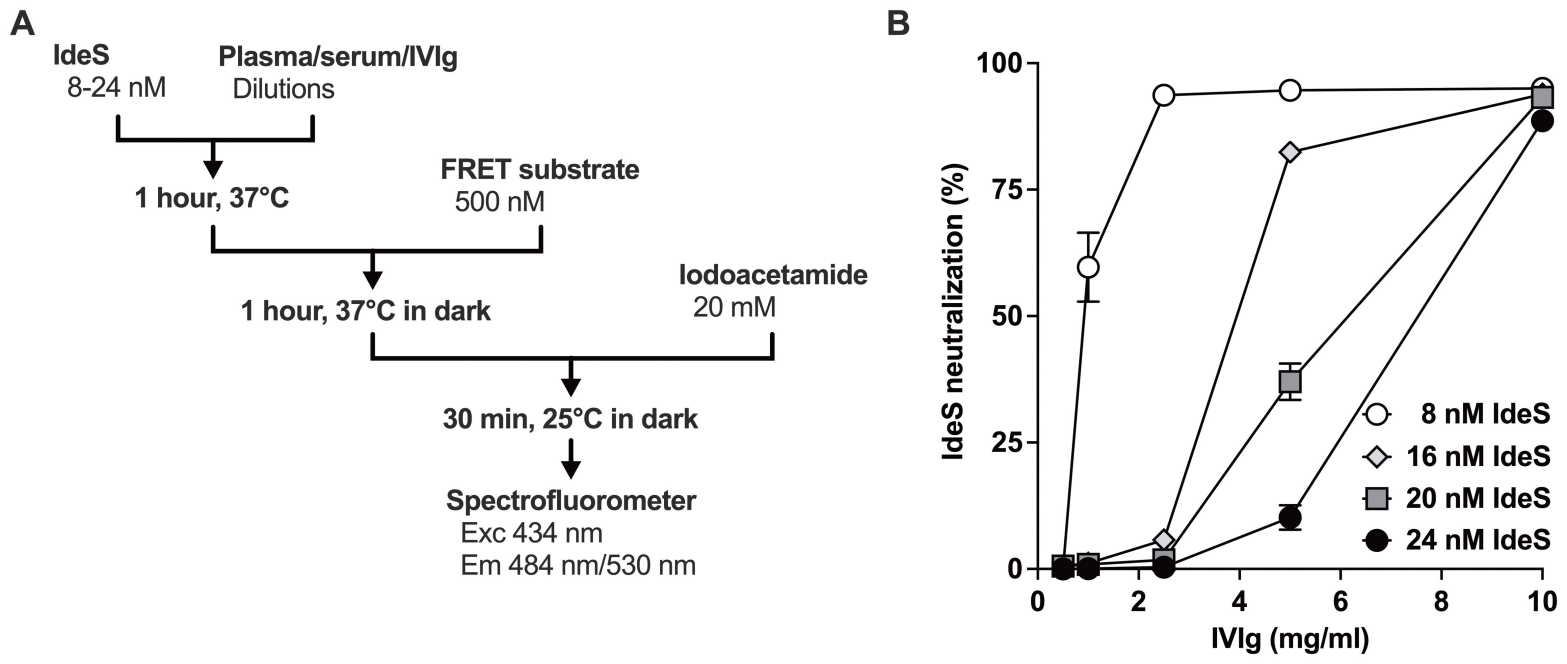
IdeS neutralization assay. **(A)** Schematic illustration of the IdeS neutralization assay. IdeS and antibody-containing preparations are incubated prior to addition of a synthetic FRET substrate for IdeS (as described in **Figure 2**) for an additional hour in the dark. The enzyme is then inactivated with iodoacetamide and fluorescence is read. **(B)** Neutralization of IdeS by IVIg. IVIg (0, 2.5, 5, 10 mg/ml) was incubated alone or with IdeS (8, 16, 20 and 24 nM) for 1 hour at 37°C before addition of the FRET substrate. Following excitation at 434 nm, the ratios of fluorescence emission measured at 530 and 484 nm were used to determine the % of IdeS neutralization as compared to the conditions where IdeS and the substrate were incubated alone (0%) or where the substrate was incubated without IdeS (100%).

In order to quantify the neutralizing activity of anti-IdeS IgG in IVIg, we engineered a synthetic fluorescence resonance energy transfer (FRET) substrate for IdeS (**Figure 2A**; **Supplementary Figure S2**). The FRET substrate was cleaved by IdeS in a dose- and time-dependent manner, as shown by SDS-PAGE (**Figures 2B, C**, see **Supplementary Figure S3** for original uncropped gel image), resulting in loss of energy transfer from eCFP to Venus as measured at 530 nm and gain in emission of eCFP at 484 nm (**Figure 2D**). The ratios of fluorescence intensities F_530_/F_484_ changed from 1.63 ± 0.26 before hydrolysis to 0.68 ± 0.03 (mean ± SD, n=4) after complete cleavage. To determine the initial velocity of the reaction, IdeS was incubated with different concentrations of FRET substrate. The F_530_/F_484_ fluorescence ratio was calculated after excitation at 434 nm every 20 seconds for each substrate concentration and was translated into µM of hydrolyzed substrate using the ratiometric method (**Figure 2E**). The initial velocity V_0_ was calculated over the linear phase (i.e., 400 seconds) and plotted as a function of the initial substrate concentration (**Figure 2F**). Fitting the experimental data to the Michaelis-Menten equation allowed calculation of the V_max_ (0.012 µM/s), K_m_ (6.07 µM) and K_cat_ (12.25 s^-1^), values that are similar to that previously determined for IdeS with different assays (**Table 1**). The FRET substrate was used to develop a functional neutralization assay (**Figure 3A**): IVIg (0.5–10 mg/ml) was incubated with IdeS (8–24 nM) for 1 hour prior to addition of the substrate. IVIg neutralized IdeS in a dose-dependent manner with ≥85% neutralization at the highest IgG concentration (**Figure 3B**). Conversely, IdeS overcame the neutralizing activity in a dose-dependent manner. Calculation of the IgG concentrations leading to 50% neutralization yielded 1.4, 5.7, 7.1 and 9.1 µg/ml for 8, 16, 20 and 24 nM IdeS, respectively (mean coefficient of variation of 6.2%, ranging from 0.3 to 23.9%, depending on the enzyme and IgG concentrations used). Taken together, these data confirm that IdeS-neutralizing IgG are present in pools of normal IgG.

**Table 1:** Michaelis constant and turnover number of IdeS.

Methods	K_m_ (µM)	k_cat_ (s^-1^)	References
Surface plasmon resonance	10.1	0.02-0.12	Agniswamy et al. (5)
SDS-PAGE/Densitometry	7.2	6.8 – 18.9	Vindebro et al. (3)
FRET	6.075	12.25	This work

### 3.2 Prevalence of anti-IdeS IgG and IgA in healthy individuals

**Figure 4:**
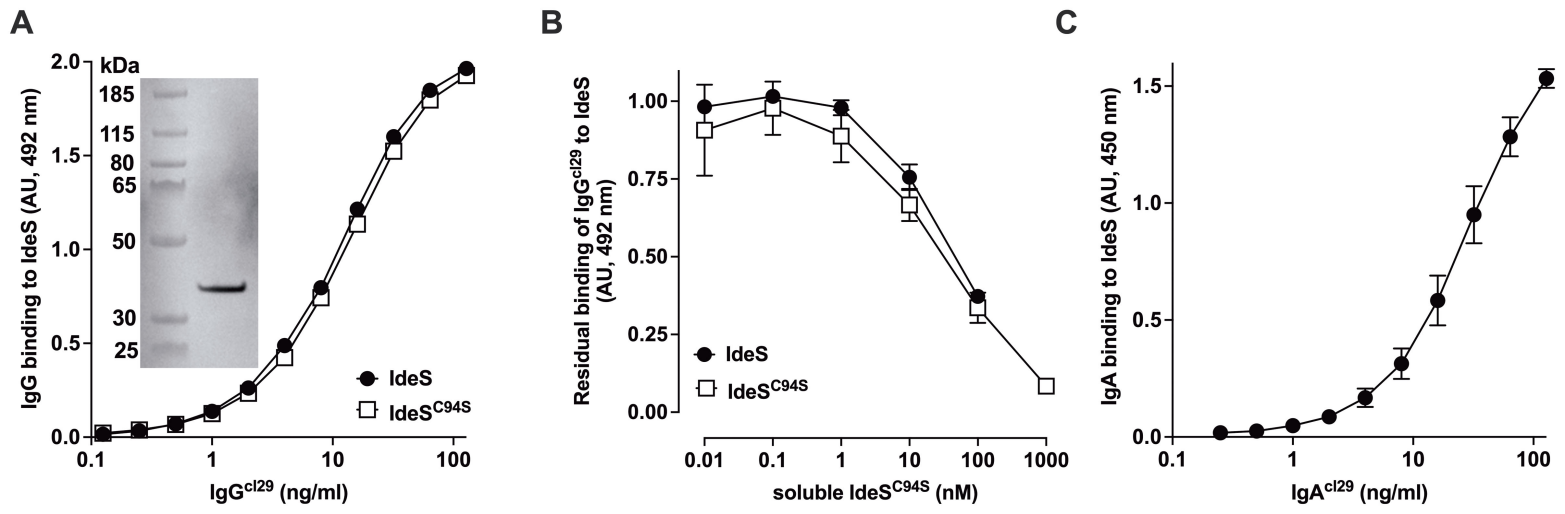
Validation of the humanized recombinant monoclonal anti-IdeS IgG^cl29^ and IgA^cl29^. **(A)** Binding of IgG^cl29^ to IdeS and to IdeS^C94S^. The humanized monoclonal anti-IdeS IgG^cl29^ was cloned from the VH and VL region genes of a mouse monoclonal anti-IdeS IgG. The human hinge region was replaced by that of mouse IgG_1_ to avoid cleave by IdeS. IgG^cl29^ was incubated in IdeS or IdeS^C94S^-coated ELISA plates. The graph depicts the levels of bound IgG, expressed in AU. Inset: the binding of anti-IdeS IgG^cl29^ to IdeS was validated by western blotting, wherein IdeS (37 kDa) was separated by 12% SDS-PAGE, blotted on nitrocellulose, incubated with IgG_1_^cl29^, prior to revelation of human IgG. **(B)** Competitive inhibition of IgG^cl29^ binding to IdeS. IgG^cl29^ (15 ng/mL) was incubated alone or with IdeS^C94S^ (0.01–1000 nM) prior to addition to IdeS or IdeS^C94S^-coated plates. Results depict the residual binding to IdeS/IdeS^C94S^ expressed in AU (means ± SD from 3 independent experiments). **(C)** Binding of IgA^cl29^ to IdeS^C94S^. The humanized monoclonal anti-IdeS IgA^cl29^ was cloned from the VH and VL region genes of a mouse monoclonal anti-IdeS IgG. IgA^cl29^ was incubated on IdeS^C94S^-coated ELISA plates. The graph depicts the levels (means ± SD from 3 independent experiments) of bound IgA, expressed in AU (optical density, 450 nm).

In order to determine the prevalence of IdeS-binding IgG and IgA under physiological conditions, we first generated humanized monoclonal anti-IdeS IgG and IgA antibodies to be used as standards in our assays. An IdeS-specific IgG-producing B-cell hybridoma (clone 29, cl29) was obtained from Ides-immunized mice, and the VL and VH genes were cloned into human IgG or IgA expression vectors. The humanized anti-IdeS IgG^cl29^ bound in a dose-dependent manner to both native IdeS and IdeS^C94S^ in an ELISA, and recognized IdeS in a western blot (**Figure 4A**, inset, see **Supplementary Figure S4** for original uncropped gel image). The binding of IgG^cl29^ was inhibited in a dose-dependent manner by soluble IdeS^C94S^ with an IC_50_ of 60.0 ± 5.7 µg/ml for IdeS and 28.5 ± 20.3 µg/ml for IdeS^C94S^ (**Figure 4B**). The recombinant anti-IdeS IgA^cl29^ bound in a dose-dependent manner to IdeS (**Figure 4C**). IgG^cl29^ was used as a standard to quantify anti-IdeS IgG in IVIg (10 mg/ml) and yielded a value of 49.6 ± 3.8 µg/ml (0.33 ± 0.03 µM, coefficient of variation: 7.6%, **Figure 1C**).

We collected plasma and serum samples from 80 and 56 healthy donors, respectively. The cohort included 64% females and 36% males, with 47 (35%) individuals between 18 and 40 years of age, 45 (33%) individuals between 41 and 60 years of age, and 44 (32%) individuals between 61 and 85 years of age (**Table 2**). Using IgG^cl29^ and IgA^cl29^ as standards, we determined the concentrations of anti-IdeS IgG and IgA in the plasma or serum of the donors by ELISA. The limit of quantification (LOQ) for the IgG and IgA ELISA was calculated using 20 blank values and the 20 serum/plasma samples with the lowest values LOQ_IgG_=2.34 µg/ml and LOQ_IgA_=0.023 µg/ml (25).

**Table 2:** Cohort of healthy individuals.

Gender	Serum	Plasma	Total
Male	14 (25.0)^†^	35 (43.8)	49 (36.0)
Female	42 (75.0)	45 (56.3)	87 (64.0)
Age groups (years)			
18-39	19 (33.9)^†^	28 (35.0)	47 (34.6)
40-59	24 (42.9)	22 (26.3)	45 (33.1)
60-85	13 (23.2)	26 (38.8)	44 (32.4)
Total	56 (41.2)	80 (58.8)	136

^†^Numbers (percentages).

**Figure 5:**
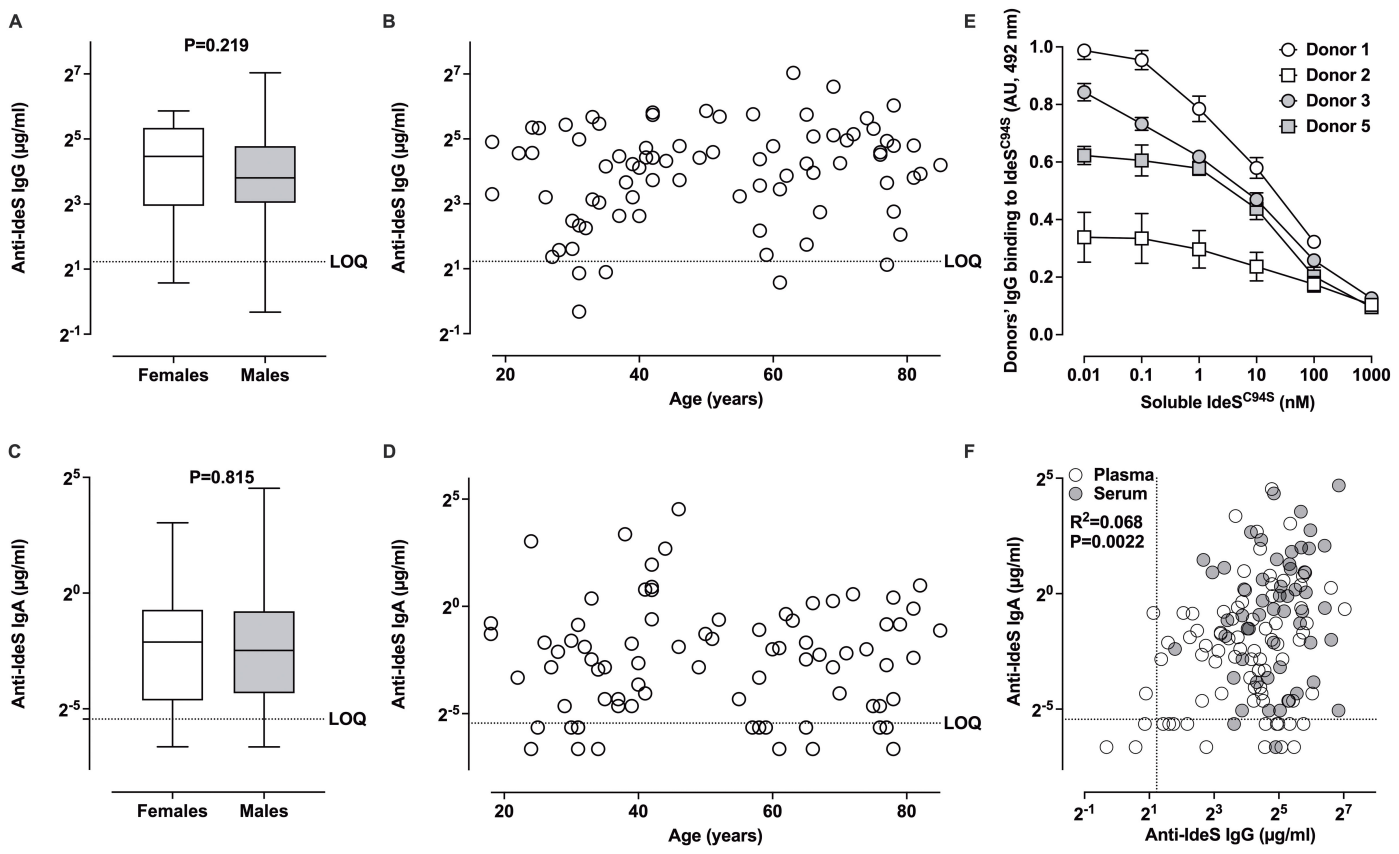
Prevalence of anti-IdeS IgG and IgA in the plasma from healthy individuals. **(A–D)**. Gender and age distribution of anti-IdeS IgG and IgA. Donors’ plasma was diluted 1/10 **(A, B)** or 1/2 **(C, D)** prior to incubation in serial dilutions on IdeS^C94S^-coated ELISA plates. Bound IgG or IgA were revealed using secondary anti-human IgG or IgA antibodies. Ig concentrations were calculated using IgG^cl29^ or IgA^cl29^ as standards. The graphs depict Ig concentrations as box and whiskers for 35 males and 45 females (empty boxes) and (full grey boxes) on the left, and the distribution of Ig concentrations as a function of age on the right. The dotted lines represent the LOQ of anti-IdeS IgG (2.34 µg/ml) and anti-IdeS IgA (0.023 µg/ml). Differences were statistically non-significant (ns) as assessed using the two-sided non-parametric Mann-Whitney test. **(E)** Competitive inhibition of IgG binding to IdeS. Plasma from 4 healthy donors was diluted to give 50% IdeS binding, and was incubated alone or with varying concentrations of IdeS^C94S^ prior to addition to IdeS^C94S^-coated plates. Results depict the residual binding to IdeS^C94S^ expressed in AU (means ± SD from 2 independent experiments). **(F)** Correlation between the levels of anti-IdeS IgG and IgA. The levels of anti-IdeS IgG measured in donors’ plasma (empty circles) and serum (full grey circles) were plotted as a function of the levels of anti-IdeS IgA. The dotted lines represent the LOQ of anti-IdeS IgG and anti-IdeS IgA. The correlation between the two sets of data was evaluated by two-tailed non-parametric Spearman correlation.

Levels of anti-IdeS IgG and IgA were higher when measured in serum than when measured in plasma, which is consistent with the highest IgG concentration in serum (**Supplementary Figure S5**). In plasma, healthy donors presented with heterogenous levels of anti-IdeS IgG: 23.89 ± 22.03 µg/ml [min: 0.80 µg/ml-max: 131.30 µg/ml]; and anti-IdeS IgA: 0.993 ± 3.007 µg/ml [0.010-23.160 µg/ml] (**Table 3**). In serum, levels of anti-IdeS IgG were 36.91 ± 26.00 µg/ml [3.47-115.30 µg/ml] and levels of anti-IdeS IgA were 2.201 ± 4.595 µg/ml [0.0-25.740 µg/ml]. There was no difference in anti-IdeS IgG or IgA titers between males and females, or according to the age of the donors (**Figures 5A–D**; **Supplementary Figures S6A, B**). Five healthy donors (3 females and 2 males) had anti-IdeS IgG levels below the LOQ_IgG_, and 20 donors (13 females and 7 males) had anti-IdeS IgA levels below the LOQ_IgA_ (**Table 3**). Hence, the prevalence of anti-IdeS IgG and IgA were 96.3% and 85.3% in our cohort, respectively. In the case of IgG, and as seen previously for IVIg and IgG^cl29^, the binding of IgG to immobilized IdeS^C94S^ was inhibited in a dose-dependent manner in the presence of soluble IdeS^C94S^, with IC_50_ of 19.1, 10.3, 12.0 and 24.2 µg/ml, for donors 1, 2, 3 and 5, respectively (**Figure 5E**). Combining data from plasma and serum samples revealed a weak but statistically significant positive correlation between the levels of anti-IdeS IgG and IgA (R^2^ = 0.068, P = 0.0022, **Figure 5F**).

**Table 3:** Levels of anti-IdeS IgG in healthy blood donors.

Samples	Nb	IgG (µg/ml)^†^	min	max	IgA (µg/ml)^†^	min	max
Plasma							
Females	45	7.95 ± 4.31	0.80	14.69	0.586 ± 1.582	0	10.28
Males	35	21.37 ± 3.74	15.16	27.78	1.681 ± 4.113	0.01	23.16
Serum							
Females	42	40.72 ± 8.96	28.62	56.31	1.723 ± 3.687	0	20.27
Males	14	83.02 ± 24.73	57.02	131.30	3.221 ± 6.787	0.03	25.74
Prevalence*		131/136>LOQ_IgG_ 96.3%			116/136>LOQ_IgA_ 85.3%		

^†^Mean ± SD; *LOQ_IgG_ = 2.34 µg/ml and LOQ_IgA_ = 0.023 µg/ml.

**Figure 6:**
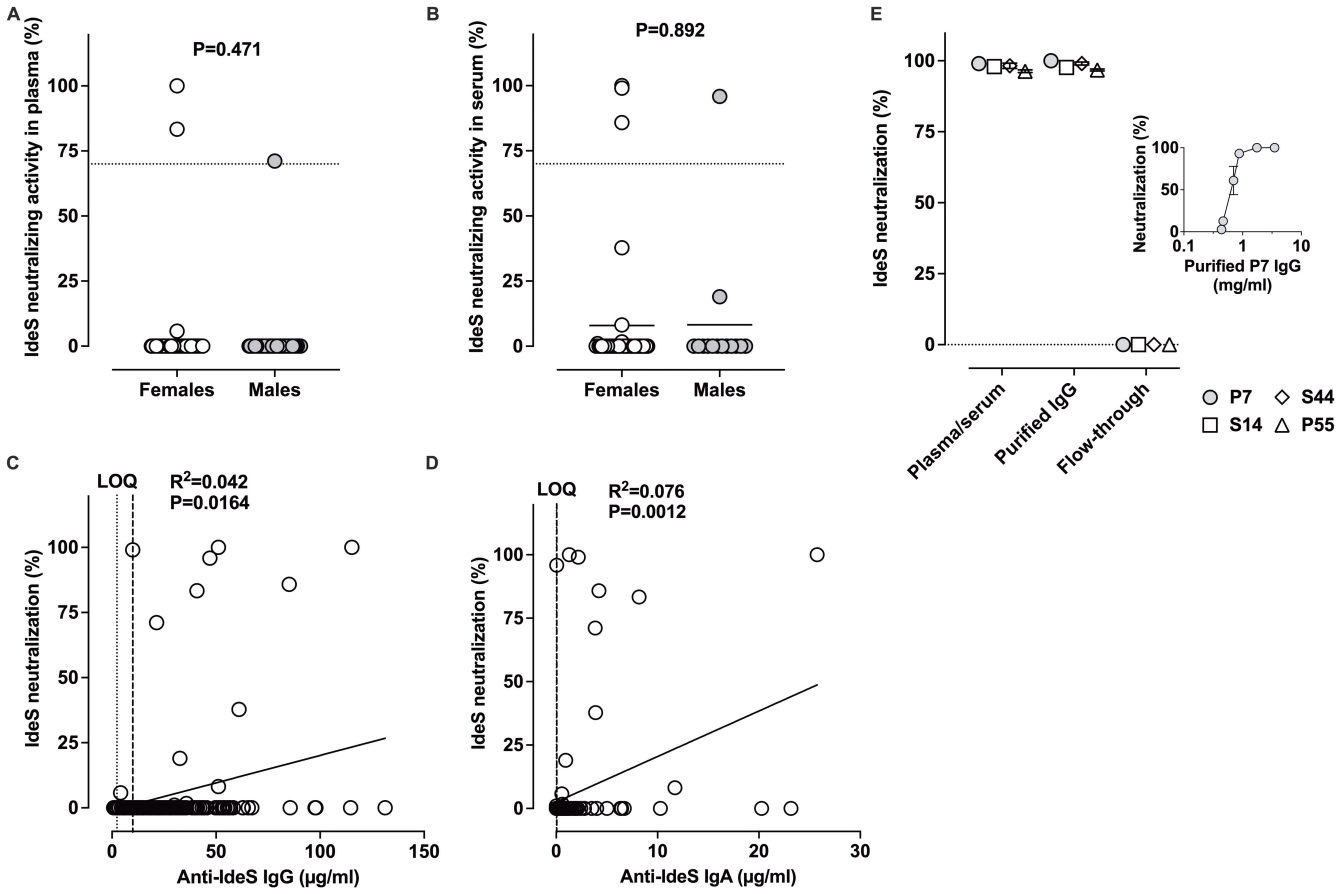
Blood from healthy donors contains IdeS-neutralizing IgG. **(A, B)**. Gender distribution of IdeS neutralizing activity. Plasma **(A)** and serum **(B)** samples (diluted 1/2.5) were incubated with 24 nM IdeS for 1 hour at 37°C and then mixed with the FRET substrate for an additional hour. Following excitation at 434 nm, the ratios of fluorescence emissions measured at 530 and 484 nm were used to determine the residual percentage of IdeS activity as compared to the conditions where IdeS was incubated alone (100%) or when the substrate was incubated without IdeS (0%). **(C)** Correlation between the levels of anti-IdeS IgG and IdeS neutralizing activity. The correlation between the two sets of data was evaluated by two-tailed non-parametric Spearman correlation. The dotted line depicts the LOQ of anti-IdeS IgG (2.34 µg/ml). The dashed line represents the lowest anti-IdeS IgG concentration (9.95 µg/ml) yielding >70% IdeS neutralizing activity. **(D)** Correlation between the levels of anti-IdeS IgA and IdeS neutralizing activity. The dotted line depicts the LOQ of anti-IdeS IgA (0.023 µg/ml). The dashed line represents the lowest anti-IdeS IgA concentration (0.05 µg/ml) yielding >70% IdeS neutralizing activity. **(E)** IdeS neutralization depends on IgG. IgG was purified from the plasma and serum from 4 donors with detectable IdeS neutralizing activity. Plasma/serum (diluted 1/10), purified IgG (1 mg/ml), and the IgG-depleted fractions (flow-through, diluted 1/10) were analyzed for their ability to neutralize IdeS. Samples were pre-incubated for 1 hour with 8 nM IdeS, prior to addition of the FRET substrate for an additional hour. The ratios of fluorescence measured at 530 and 484 nm after excitation at 434 nm, were used to determine the percentage of IdeS neutralization, as described previously. The inset depicts IdeS (8 nM) neutralization as a function of the concentration of IgG (0.4–5 mg/ml)) purified from the plasma of donor 7.

We then investigated the presence of IdeS neutralizing antibodies in the plasma and sera from healthy donors. In this assay, we first used a concentration of 24 nM IdeS and a plasma/serum dilution of 1:2.5. Under such experimental conditions, 3 out of 80 plasma (2 females, 1 male, 3.75%) and 4 out of 56 sera (3 females, 1 male, 7.14%) had IdeS neutralizing activities >70% (**Figures 6A, B**: above the dotted line). IdeS neutralizing activity above 70% was detected for samples that had anti-IdeS IgG≥9.95 µg/ml or IgA≥0.05 µg/ml (**Figures 6C, D**), although most samples with IgG/IgA concentrations greater than the latter thresholds did not show IdeS neutralization, at least in the present experimental conditions. Of note, with a serum/plasma dilution of 1:10, only one sample showed IdeS neutralizing activities >70%. There was a positive correlation, albeit with poor goodness-of-fit, between the IdeS neutralizing activity and the levels of anti-IdeS IgG (R^2^ = 0.042, P = 0.0164), or of anti-IdeS IgA (R^2^ = 0.076, P = 0.0012).

To investigate whether the IdeS neutralizing activity is carried by the IgG or IgA fraction of plasma/serum, we purified IgG from 2 serum and from 2 plasma samples on protein G-agarose beads. The purification of the IgG and depletion from plasma/serum was confirmed by SDS-PAGE (**Supplementary Figure S7**). The starting material (plasma/serum), purified IgG and IgG-depleted fractions (flow-through) were then assessed for IdeS neutralizing activity using 8 nM IdeS. While IdeS neutralization was readily detected in plasma/serum and in the purified IgG fractions of the four donors, it was completely absent from the flow-through (**Figure 6E**). IgG-mediated neutralization of IdeS was dependent on IgG concentration (**Figure 6E**, inset).

## 4 Discussion

Here, we describe the prevalence of anti-IdeS IgG and IgA, and of IdeS-neutralizing antibodies, in a cohort of healthy adult donors. To quantify anti-IdeS IgG, we developed an in-house ELISA using the recombinant humanized anti-IdeS IgG^cl29^ as a standard. IgG^cl29^ does not neutralize IdeS, and its epitope specificity remains unknown. The fact that IgG^cl29^ bound IdeS through its Fab fragments and not as a substrate, is demonstrated by the strong binding detected by ELISA at concentrations well below the dissociation constant previously reported for the IgG1-IdeS complex (K_D_=2.5 µM) (5), and by the lack of binding to IdeS of several unrelated human monoclonal IgG_1_ under similar conditions. The LOQ determined for our assay (2.34 µg/ml) was similar to the cutoff determined by Winstedt et al. (10) using a formerly available commercially assay, i.e., 2 µg/ml. Using our assay, we detected anti-IdeS IgG at a concentration of 49.6 ± 3.8 µg/ml in a 10 mg/ml IVIg preparation, as well as in more than 95% of the individuals included in the study, with mean concentrations of 23.9 ± 22.0 µg/ml in plasma and 36.9 ± 26.0 µg/ml in serum. The presence of anti-IdeS IgG in healthy individuals had already been reported in several studies (7, 19, 20). Indeed, using a cohort size (i.e., reference cohort of 130 individuals) close to the one included in the present study, Winstedt et al. (10) reported a similar prevalence and inter-individual variability of anti-IdeS IgG. The presence of anti-IdeS IgG in healthy individuals is thought to result from previous infections by *S. pyogenes* (19). The anti-IdeS IgG concentrations we determined are 2-3-fold higher than the one calculated by Winstedt et al. (10) (12.9 ± 13.3 µg/ml, p<0.0001) using sera from 208 healthy male donors, and below values determined in sera from 40 healthy donors by Leborgne et al. (18) (100.5 ± 133.8 µg/ml) who used IVIg as a standard.

To our knowledge, evidence for the existence of anti-IdeS antibodies with non-IgG isotypes in humans is lacking. Lorant et al. (20) stated that, out of 102 patients included in their study, none tested positive for IgE against IdeS prior to enrolment. Likewise, Lonze et al. (21) mentioned that the presence of anti-IdeS IgE was an exclusion criterium in their trial, but did not indicate the detection assay and the number of patients who tested positive. Infection by *S. pyogenes* requires contact with skin or mucosa, with respiratory transmission being the most frequent route of infection (30). Because IgA is the primary antibody associated with mucosal immunity, we investigated the presence of anti-IdeS IgA in healthy donors. Interestingly, IgA is not a substrate for IdeS. Hence, putative anti-IdeS IgA are not eliminated upon administration of therapeutic IdeS, in contrast to anti-IdeS IgG. We thus developed an in-house anti-IdeS IgA ELISA using the recombinant humanized IgA^cl29^ as a standard. In our cohort, more than 80% of the individuals had levels of anti-IdeS IgA above the LOQ, with mean concentrations of 0.99 ± 3.01 µg/ml in plasma and 2.20 ± 4.60 µg/ml in serum. Of note, IgA^cl29^ shares identical VH and VL regions as IgG^cl29^, thus allowing for a direct comparison between the levels of anti-IdeS IgG and IgA. Despite large variations in anti-IdeS IgG and IgA concentrations among donors, there was a significant correlation between the two values. Yet, the ratio of anti-IdeS IgG over anti-IdeS IgA in serum, equal to 16, was greater than that (or on the high range of) the total serum IgG/IgA ratio, assuming concentrations of 7–16 mg/ml IgG and 0.7–4 mg/ml IgA. Whether this reflects the fact that serum IgG immune responses predominate over mucosal IgA responses in *S. pyogenes* infections (31), at least in the pharyngitis context, remains to be demonstrated.

Therapeutic IdeS (Idefirix^®^) was approved in the EU in 2020 to remove donor specific antibodies in highly sensitized adults requiring kidney transplantation (11) and was tested in other clinical situations, notably in patients with anti-glomerular basement membrane antibody disease (NCT03157037) or Guillain-Barré Syndrome (NCT03943589). The presence of pre-existing anti-IdeS antibodies in patients treated with IdeS may have different consequences including accelerated IdeS elimination, increased risk of immune complex-mediated hypersensitivity or infusion-like reactions, as shown in patients with thrombotic thrombocytopenic purpura (28), and neutralization leading to reduction of IdeS efficacy. The existence of IdeS neutralizing antibodies was demonstrated in acute-phase infected patients with pharyngotonsillitis, sepsis, erysipelas or invasive *S. pyogenes* infections, with a prevalence of ~71% (19). Accordingly, we had suggested the onset of IdeS neutralizing antibodies in non-human primates following intravenous injection of IdeS (18). Conversely, the presence of anti-IdeS IgG in healthy individuals was reported to not affect the cleavage of IgG by IdeS, even in the sera with the highest titers against IdeS (7). The presence of IdeS neutralizing antibodies was nevertheless suggested in therapeutic pools of normal IgG (22). In all the latter studies, IdeS-mediated IgG cleavage and neutralization thereof, were always studied by SDS-PAGE because no surrogate substrate for IdeS was available at the time when the studies were performed (32).

We describe here a quantitative assay for the detection of IdeS neutralizing antibodies. The assay relies on a synthetic substrate for IdeS that was engineered based on the structure of the human IgG_1_ and that exploits the FRET technology (**Figure 2A**). Cleavage of IgG by IdeS generates a F(ab’)_2_ fragment wherein the two Fabs remain associated by inter-chain disulfide bonds at the level of the hinge region. To ensure that cleavage of the FRET substrate by IdeS frees the fluorochromes from each other and abrogates the FRET signal, we only cloned the lower part of the hinge region that contains the APELLG amino-acids, and excluded amino-acids that compose the upper (EPKSCDKTHT) and core (CPPCP) hinge. Importantly, our synthetic FRET substrate for IdeS only mimics the first cleavage of the IgG molecule as can be inferred from its structure, and is confirmed by the calculated kinetic parameters (**Table 1**). The assay is also very sensitive to small changes in enzyme or IgG concentrations (**Figure 3B** and inset of **Figure 6E**). It however presents several advantages over existing methods such as SDS-PAGE, surface plasmon resonance or LC-MS, including simple and rapid use, variety of biological fluids in which the IdeS proteolytic activity may be measured, quantitative nature and medium to high-throughput capacity. The neutralization assay was validated using therapeutic pools of human IgG (IVIg), confirming the presence of IgG with neutralizing activity towards IdeS (22). The level of IdeS neutralization depended on the amount of IdeS used in the assay. The C_max_ calculated for IdeS concentration in serum during clinical trials was 8.3 ± 3.7 µg/ml (224 ± 100 nM) for 0.25 mg IdeS/kg body weight (10, 20). Thus, under clinical conditions, the IgG/IdeS ratio is ~13 mg/ml IgG versus 224 nM IdeS. In our assay, at 1 mg/ml, IVIg neutralized IdeS by ~60% only at an IdeS concentration of 8 nM, i.e., an IgG/IdeS ratio 2–3 times greater than the one achieved during IdeS treatment. IdeS neutralizing antibodies were also detected in 7/136 the healthy donors upon incubation of plasma/serum at 1:2.5 dilution with IdeS at 24 nM. When the clinically relevant IgG/IdeS ratio was used in the assay (i.e., plasma/serum at 1:10 dilution with IdeS at 24 nM), only one individual showed >70% IdeS neutralization. The fact that most individuals in our cohort have anti-IdeS IgG while only 1% exhibit IdeS neutralizing activity, suggest differences in epitope specificity of anti-IdeS IgG among individuals, with a minority of them recognizing the catalytic site or substrate binding domains of IdeS. This is strengthened by the positive but poor correlation between the IdeS neutralizing activity and the levels of anti-IdeS IgG (**Figure 5F**), and consistent with observations during acute *S. pyogenes* infections, where neutralizing antibodies specifically mapped to the catalytic domain, unlike the predominant non-neutralizing specificities seen in healthy donors (19). Importantly, the neutralizing activity resided in the IgG fraction of the serum/plasma. This does not exclude a potential neutralizing activity of anti-IdeS IgA, that may however be too poorly concentrated in serum/plasma to be detected with our assay, and/or to exert any biological activity.

Taken together, the results indicate that anti-IdeS IgG and anti-IdeS IgA are present in a large majority of healthy individuals. The presence of circulating anti-IdeS antibodies however translates into *in vitro* detectable IdeS neutralizing activity in about 1% of the normal population. Whether the prevalence of IdeS neutralizing antibodies is the same among individual from different geographical areas or increases in some pathologies, such as chronic kidney disease, remains to be determined. Importantly, few examples of patients who do not respond adequately to IdeS treatment have been mentioned. Taken together, these observations underscore the need for a prospective clinical trial to assess IdeS-binding and IdeS-neutralizing antibody levels in highly sensitized kidney transplant recipients, both prior to and following IdeS administration. In this respect, the assays validated in the present work represent indispensable tools. In the future, our novel functional IdeS neutralization assay may serve as a predictive tool to aid nephrologists in determining patient eligibility for IdeS-based desensitization protocols.

## Data Availability

The original contributions presented in the study are included in the article/**Supplementary Material**. Further inquiries can be directed to the corresponding author.
