# Supplementary material for: Novel assays to assess the prevalence and neutralizing potential of anti-IdeS antibodies in healthy humans
Source: Front Immunol. 2026 Feb 9;17:1728855. doi: 10.3389/fimmu.2026.1728855 (PMC12926173; doi:10.3389/fimmu.2026.1728855)
Supplement: Supplementary file 1 [file DataSheet1.docx]

**Supplemental information**

**Supplementary Figure S1**


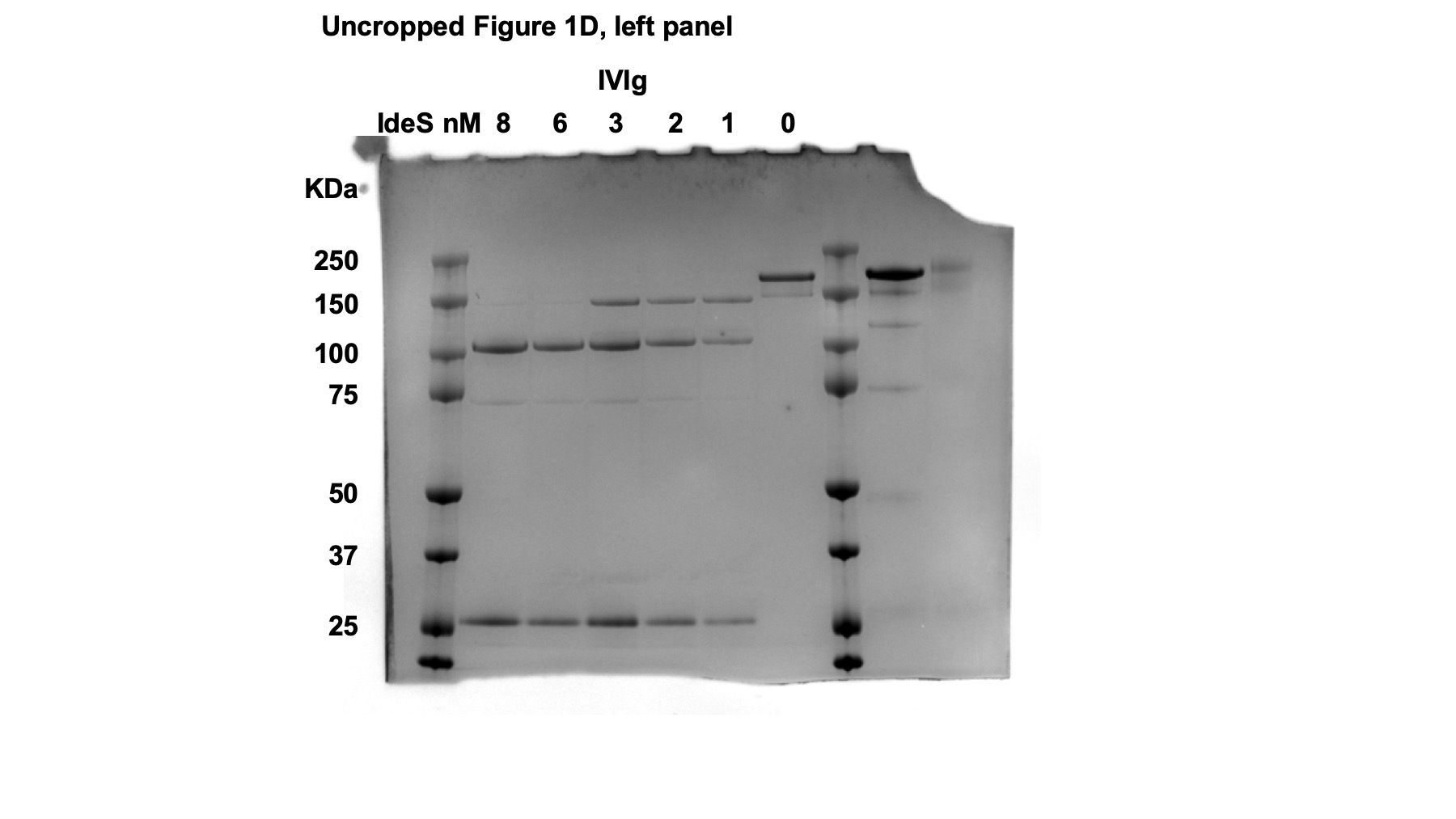

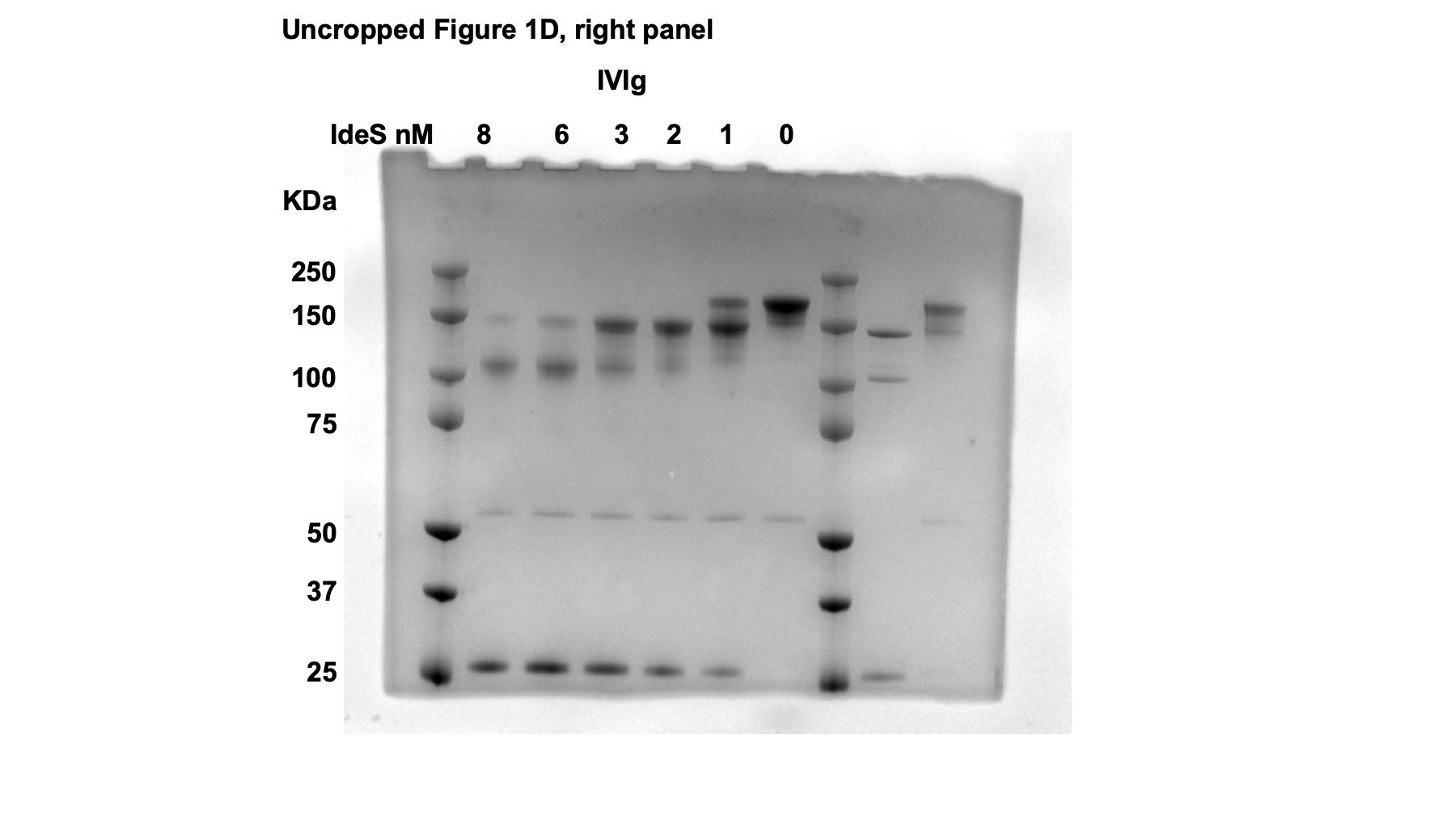

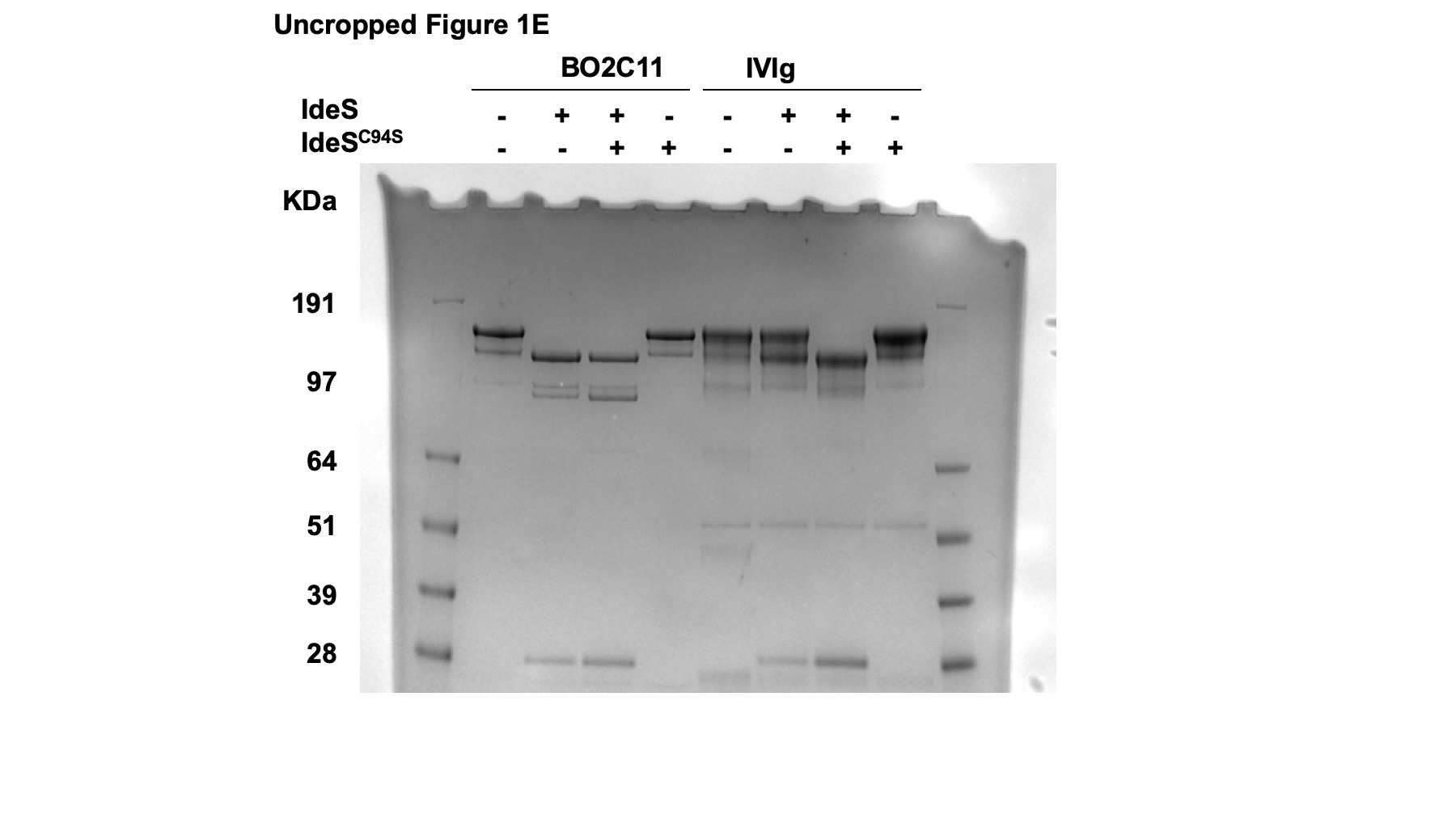


**Figure S1. Original gels for Fig 1D and 1E.** For the legends, please refer to the legends of Figures 1D and 1E.

**Figure S2. Synthetic FRET substrate for IdeS.** The heterodimeric FRET substrate (structure in **Figure 2A**) and the individual the eCFP-Fc and Venus-Fc monomers were produced using the Expi293 technology (ThermoFisher) and purified from supernatant by cobalt (FRET substrate and eCFP-Fc) or protein G (Venus-Fc) affinity chromatography and size-exclusion chromatography. **A.** The graph depicts the elution profile of the FRET substrate by size-exclusion chromatography (1 ml/min) with a single peak at 170 ml, detected by absorbance at 280 nm (plain line curve), 434 nm (dotted line curve) and 515 nm (dashed line curve). **B.** The purified FRET substrate and the individual eCFP-Fc and Venus-Fc monomers (5 µg/lane) were separated by SDS-PAGE in NuPAGE 4-12% gradient Bis-Tris protein gels (Thermo Scientific) under non-reducing conditions, and revealed using Coomassie blue staining. The molecular weight standard is shown on the left. **C.** The fluorescence emission profiles of the purified FRET substrate and individual monomers were recorded after excitation at 434 nm (eCFP-Fc monomer and substrate) or at 480 nm (Venus-Fc).

**Supplementary Figure S3**

**
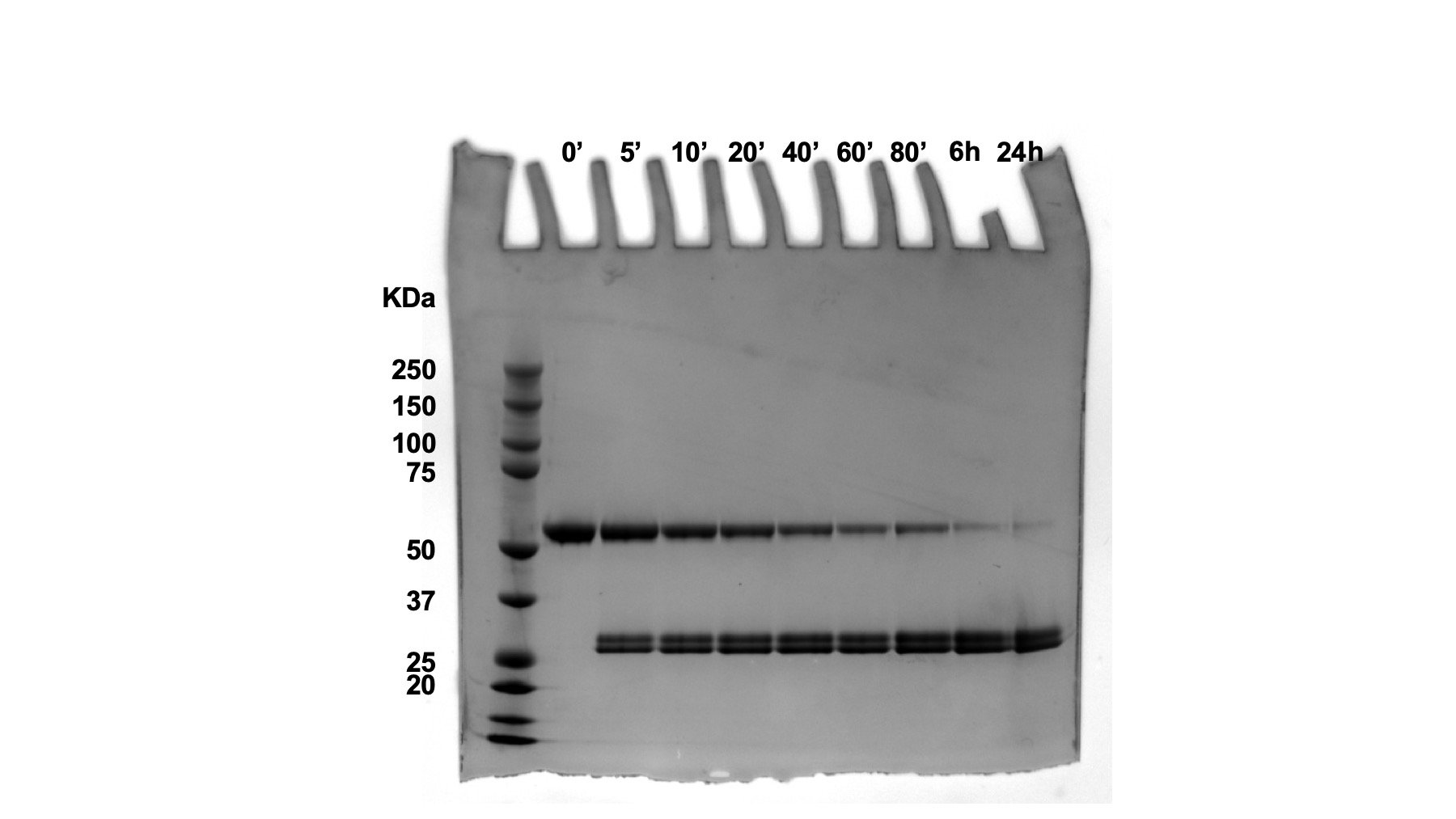
**

**Figure S3. Original gels for Fig 2C.** For the legend, please refer to the legend of Figure 2C.

**Supplementary Figure S4**

**
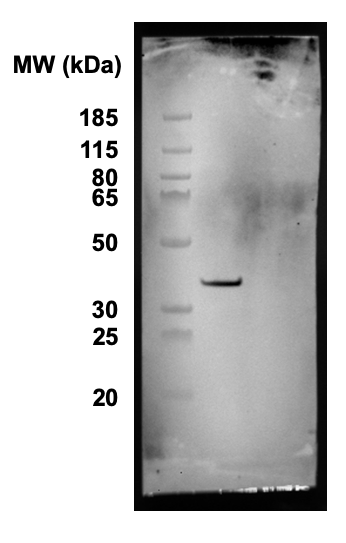
**

**Figure S4. Original gels for Fig 4A.** For the legend, please refer to the legend of Figure 4A (inset).

**Figure S5.** **Anti-IdeS IgG and IgA in the plasma and serum from healthy individuals.** The plasma (blue columns) our serum (green columns) from healthy individuals was diluted 1/10 (**A**) or 1/2 (B) prior to incubation in serial dilutions on IdeS^C94S^-coated ELISA plates. Bound IgG (**A**) or IgA (**B**) were revealed using secondary anti-human IgG or IgA antibodies. Ig concentrations were calculated using IgG^cl29^ or IgA^cl29^ as standards. The graphs depict Ig concentrations as box and whiskers for 80 plasma (blue) and 56 sera (green). The dotted lines represent the LOQ of anti-IdeS IgG (2.34 µg/ml) and anti-IdeS IgA (0.023 µg/ml). Statistically differences were assessed using the two-sided non-parametric Mann-Whitney test.

**Figure S6. Prevalence of anti-IdeS IgG and IgA in the serum from healthy individuals. A-B.** **Gender and age distribution of anti-IdeS IgG and IgA.** Donors’ serum was diluted 1/10 (**A**) or 1/2 (**B**) prior to incubation in serial dilutions on IdeS^C94S^-coated ELISA plates. Bound IgG or IgA were revealed using secondary anti-human IgG or IgA antibodies. Ig concentrations were calculated using IgG^cl29^ or IgA^cl29^ as standards. The graphs depict Ig concentrations as box and whiskers for 35 males and 45 females (blue) and (green) on the left, and the distribution of Ig concentrations as a function of age on the right. The dotted lines represent the LOQ of anti-IdeS IgG (2.34 µg/ml) and anti-IdeS IgA (0.023 µg/ml). Differences were statistically non-significant (ns) as assessed using the two-sided non-parametric Mann-Whitney test.

**Figure S7. Migration profiles of plasma/serum and corresponding purified IgG.** IgG was purified from the plasma (P7 and P55) and serum (S14, S44) of healthy individuals. Plasma/serum and the corresponding purified IgG (5 µg/lane) were separated by 12% SDS-PAGE under non-reducing conditions, and revealed using Coomassie blue staining. Molecular weight markers are shown on the left of the gel.
